# 2,3,5,4′-Tetrahydroxystilbene-2-O-*β*-glucoside Isolated from Polygoni Multiflori Ameliorates the Development of Periodontitis

**DOI:** 10.1155/2016/6953459

**Published:** 2016-07-18

**Authors:** Yu-Tang Chin, Meng-Ti Hsieh, Chi-Yu Lin, Po-Jan Kuo, Yu-Chen S. H. Yang, Ya-Jung Shih, Hsuan-Yu Lai, Guei-Yun Cheng, Heng-Yuan Tang, Chen-Chen Lee, Sheng-Yang Lee, Ching-Chiung Wang, Hung-Yun Lin, Earl Fu, Jacqueline Whang-Peng, Leroy F. Liu

**Affiliations:** ^1^Taipei Cancer Center, Taipei Medical University, 250 Wu-Hsing Street, Taipei 11031, Taiwan; ^2^Department of Dentistry, Wan-Fang Medical Center, Taipei Medical University, 250 Wu-Hsing Street, Taipei 11031, Taiwan; ^3^PhD Program for Cancer Biology and Drug Discovery, College of Medical Science and Technology, Taipei Medical University, 250 Wu-Hsing Street, Taipei 11031, Taiwan; ^4^Department of Periodontology, School of Dentistry, National Defense Medical Center and Tri-Service General Hospital, Taipei, Taiwan; ^5^Graduate Institute of Life Sciences, National Defense Medical Center, Taipei, Taiwan; ^6^Joint Biobank, Office of Human Research, Taipei Medical University, 250 Wu-Hsing Street, Taipei 11031, Taiwan; ^7^Pharmaceutical Research Institute, Albany College of Pharmacy and Health Sciences, Albany, NY, USA; ^8^Department of Microbiology and Immunology, School of Medicine, China Medicine University, Taichung, Taiwan; ^9^School of Dentistry, College of Oral Medicine, Taipei Medical University, 250 Wu-Hsing Street, Taipei 11031, Taiwan; ^10^School of Pharmacy, College of Pharmacy, Taipei Medical University, 250 Wu-Hsing Street, Taipei 11031, Taiwan

## Abstract

Periodontitis, a chronic infection by periodontopathic bacteria, induces uncontrolled inflammation, which leads to periodontal tissue destruction. 2,3,5,4′-Tetrahydroxystilbene-2-O-beta-glucoside (THSG), a polyphenol extracted from Polygoni Multiflori, reportedly has anti-inflammatory properties. In this study, we investigated the mechanisms of THSG on the* Porphyromonas gingivalis*-induced inflammatory responses in human gingival fibroblasts and animal modeling of ligature-induced periodontitis. Human gingival fibroblast cells were treated with lipopolysaccharide (LPS) extracted from* P. gingivalis* in the presence of resveratrol or THSG to analyze the expression of TNF-*α*, IL-1*β*, and IL-6 genes. Increased AMP-activated protein kinase (AMPK) activation and SirT1 expression were induced by THSG. Treatment of THSG decreased the expression of LPS-induced inflammatory cytokines, enhanced AMPK activation, and increased the expression of SirT1. In addition, it suppressed the activation of NF-*κ*B when cells were stimulated with* P. gingivalis* LPS. The anti-inflammatory effect of THSG and P. Multiflori crude extracts was reproduced in ligature-induced periodontitis animal modeling. In conclusion, THSG inhibited the inflammatory responses of* P. gingivalis*-stimulated human gingival fibroblasts and ameliorated ligature-induced periodontitis in animal model.

## 1. Introduction

Periodontitis is an immune-associated inflammatory disease of the periodontium characterized by progressive destruction of the supporting tissues of teeth [1]. It can destroy the surrounding connective tissue and adjacent alveolar bone and eventually causes tooth loss [2, 3]. The immune and inflammatory responses that are critical to the pathogenesis of periodontitis are modified by a number of host-related factors [4]. Therefore, while microbial plaque is the primary etiology and initiates the host immune response, which induces the signs of periodontitis [5], the major component of the outer membrane of Gram-negative bacteria,* Porphyromonas gingivalis*, lipopolysaccharide (LPS), initiates the production of various cytokines such as interleukin-8 (IL-8) and TNF-*α* [6] which infiltrate gingival connective tissue and elicit a local inflammatory response. It further causes an increase in number and activity of polymorphonucleocytes (PMN) along with these cytokines. PMNs also produce reactive oxygen species (ROS) superoxide via the respiratory burst mechanism as part of the defense response to infection [7]. The neutrophil-produced high levels of ROS are associated with the local and systemic destructive phenotype found in the chronic inflammatory disease periodontitis [8]. The ROS has the capacity to activate macrophages to synthesize and secrete inflammatory cytokines and hydrolytic enzymes, which manifest potent proinflammatory and catabolic activities and play key roles in periodontal tissue breakdown [9]. Periodontitis has been shown to link other chronic diseases such as diabetes, cardiovascular diseases, and rheumatoid arthritis. Studies indicate that treatment of rheumatoid arthritis may improve the associated periodontal disease and vice versa [8].

Lately natural compounds capable of modulating the host inflammatory response have received considerable attention [10]. Naturally occurring orally related antibacterial and anti-inflammatory actions of polyphenols are capable of modulating the host inflammatory response. Of these, certain flavonoids appear to stand out because of their beneficial profile and clinical evidence [10]. Resveratrol (trans-3,4′,-5-trihydroxystilbene) is a widely existing stilbenoid in grape skin [11] and in other plants such as berries and peanuts [12, 13]. It exhibits antioxidant activity and contributes to a reduction in inflammation [14]. Resveratrol has been shown to neutralize free radicals and other oxidizing molecules [13]. Its supplementation directly suppresses the release of proinflammatory cytokines such as TNF-*α*, IL-1*β*, IL-6, IL-10, MCP-1, IFN*α*, and IFN*β* in a wide range of tissues [15–19], including the brain in rodents [20]. It has been shown to significantly inhibit NF-*κ*B-dependent cell adhesion molecules in monocyte adhesion to the endothelium induced by* P. gingivalis *LPS suggesting that resveratrol has therapeutic effect in periodontal pathogen-induced vascular inflammation [21]. Resveratrol has been shown to effectively delay osteoblastogenesis and subsidize new bone formation [22]. These properties are very essential for the treatment of periodontitis.

The stilbene glucoside 2,3,5,4′-tetrahydroxystilbene-2-O-*β*-D-glucoside (THSG) is one of the major bioactive components of* Polygonum multiflorum* Thunb. (He Shou Wu), which has been shown to exert antiaging activities [23]. It also has protective effects on experimental colitis by reducing the level of oxygen and nitrogen free radicals [24]. Mechanism involved in* P. multiflorum*-induced antiatherosclerosis may be caused by THSG-induced antagonistic effects on oxidation of lipoprotein, proliferation, and decrease of NO content of coronary arterial smooth muscle cells [25]. It also has been shown to exert protective effect on cardiotoxicity induced by doxorubicin* in vitro* and* in vivo* [26]. In addition, THSG can diminish peroxidation levels in the brain in a mouse model of Alzheimer's disease or cerebral ischemia-reperfusion. The protective effects of THSG are mediated by modulation of JNK, SirT1, and NF-*κ*B pathways [27].

In animals, experimental periodontitis can be induced by dietary manipulation [28], bacterial toxin injection [29], laying of peridental silk ligatures or orthodontic elastics for bacterial colonization [29, 30], and surgical removal of alveolar bone [31, 32]. In the present study, we investigate the role of ethanol extract of P. Multifloriand pure 2,3,5,4′-tetrahydroxystilbene-2-O-beta-glucoside on the degree of alveolar bone loss associated with periodontitis in rats and its signal transduction pathways involved in the preventative and inhibitory effects in the development of periodontitis.

## 2. Materials and Methods

### 2.1. Reagents

2,3,5,4′-Tetrahydroxystilbene-2-O-beta-glucoside (THSG, purity: 95%, dissolved in 50% ethanol for animal feeding; dissolved in DMSO for cell treatment) was kindly provided by Dr. Ching-Chiung Wang. Resveratrol (Sigma-Aldrich, Inc., St. Louis, Mo, USA) was dissolved in 50% ethanol for animal feeding and dissolved in DMSO for cell treatment.

### 2.2. Plant Materials

*P*.* multiflora* Thunb was purchased from a traditional Chinese medicine market in China and identified by Industrial Technology Research Institute, Taiwan. Moreover, voucher specimens (He Shou Wu 01) of dried rhizoma were deposited in the Graduate Institute of Pharmacognosy, College of Pharmacy, Taipei Medical University, Taipei, Taiwan.

### 2.3. Extraction

The rhizome of* P. multiflora* was cut into small pieces, immersed in a 10-fold amount of 50% ethanol, and refluxed for 2 hour at 65°C, twice. And then the twice filtrate was combined and concentrated under a vacuum using a rotary evaporator to remove the ethanol. The ethanolic extract was freeze-dried to yield the powder (PM-E). The yield of PM-E was about 27.82% (w/w) and 2,3,5,4′-tetrahydroxystilbene-2-O-beta-glucoside (THSG) amount is 8.0% in PM-E by HPLC analysis. The substance marker, THSG (purity: 95%), was kindly provided by Dr. Ching-Chiung Wang.

### 2.4. Isolation and Culture of Human Gingival Fibroblasts

Human gingival fibroblasts (HGFs) were obtained from five patients (males aged 22 to 32 years; mean age: 24 years), and all experiments were repeated three times as described in our previous study [33]. Briefly, gingival specimens were taken from gingivectomy, crown lengthening, or distal wedge procedure of noninflamed periodontal tissues. Specimens were directly submerged in Leibovitz L-15 medium (Invitrogen, Grand Island, NY, USA) containing 2 mg/mL Dispase II (Roche Diagnostics, Indianapolis, IN, USA) and 10% fetal bovine serum (FBS) (Invitrogen) at 4°C for 2 days. After separating and removing the outer epithelial layer, the connective tissue was minced and digested in Leibovitz L-15 medium containing 10% FBS and 2 mg/mL collagenase (Sigma-Aldrich, Inc.) for 24 hours. Tissue was then placed in flasks containing 10% FBS in Dulbecco modified Eagle medium/F12 (DMEM/F12) (Invitrogen) to allow the cells to migrate from the explants. HGFs of five to seven passages were used for these experiments. When HGFs reached 80% confluence, cells were starved in serum-free DMEM/F12 for 24 hours before experiments. Similar patterns were examined in different cell origins, and one of them was selected to report. Approval of all procedures was granted by the Ethics Committee of the Faculty of Medicine, Tri-Service General Hospital, Taipei, Taiwan (TSGHIRB number 2-103-05-067). All participants included in the study signed a written consent form before the procedure.

### 2.5. Cell Viability Test

To evaluate the toxic effects of resveratrol and THSG on HGFs, HGFs were placed at a density of 1 × 10^3^ cell/well in the wells of 96-well plates containing DMEM/F12 and 10% FBS and cultured until 80% confluence. The cells were washed once with phosphate-buffered saline, and the medium was replaced with serum-free medium for 24 hours' starvation. THSG and resveratrol were solubilized in DMSO at different concentrations in all experiments below. The cell viability of HGFs was tested after treatment of THSG (0, 10, 25, 100, or 200 *μ*M) or resveratrol (0, 10, 25, 100, or 200 *μ*M) in 0.25% stripped FBS medium for 72 h with reflashed medium with agents daily. The cell viability was performed with CellTiter 96® AQueous One Solution (Promega, Madison, WI, USA) according to the manufacturer's protocol.

### 2.6. Quantitative Real-Time PCR

To examine the effects of resveratrol and THSG on the mRNA expression of proinflammatory cytokines, we treated HGFs with vehicle, 1 *μ*g/mL* P. gingivalis* LPS (*Porphyromonas gingivalis* lipopolysaccharide) (InvivoGen, San Diego, CA, USA), 1 and 10 *μ*M resveratrol, and 1 and 10 *μ*M THSG or combined treatments for 6 h. Furthermore, in order to investigate the impacts of THSG on proinflammatory cytokines and inflammatory markers in ligature-induced experimental periodontitis of rats, silk-surrounded gingival tissues were removed and homogenized by a high-throughput tissue (Precellys® 24, Bertin Corp., Rockville, MD, USA). Total RNA was extracted and genomic DNA was also eliminated with illustra RNAspin Mini RNA Isolation Kit (GE Healthcare Life Sciences, Buckinghamshire, United Kingdom). 1 *μ*g of DNase I-treated total RNA was reverse-transcribed with RevertAid H Minus First Strand cDNA Synthesis Kit (Life Technologies Corporation, Carlsbad, California, USA) into cDNA and used as the template for real-time PCR reactions and analysis. The real-time PCR reactions were performed using QuantiNova*™* SYBR® Green PCR Kit (QIAGEN) on CFX Connect*™* Real-Time PCR Detection System (Bio-Rad Laboratories, Inc., Hercules, CA, USA). This involved an initial denaturation at 95°C for 5 min, followed by 45 cycles of denaturing at 95°C for 5 sec and combined annealing/extension at 60°C for 10 sec, as described in the manufacturer's instructions. The primer sequences were shown as follows:* Homo sapiens* tumor necrosis factor-alpha (*TNF-α*), forward 5′-GCCCATGTTGTAGCAAACCC-3′ and reverse 5′-TATCTCTCAGCTCCACGCCA-3′ (accession number NM_000594.3);* Homo sapiens* interleukin-1 beta (*IL-1β*), forward 5′-CTTCGAGGCACAAGGCACA-3′ and reverse 5′-GCTTCAGACACTTGAGCAATGA-3′ (accession number NM_000576.2);* Homo sapiens* interleukin-6 (*IL-6*), forward 5′-AATCATCACTGGTCTTTTGGAG-3′ and reverse 5′-GCATTTGTGGTTGGGTCA-3′ (accession number NM_000600.3);* Homo sapiens* sirtuin 1 (*SirT1*), forward 5′-TTGGGTACCGAGATAACCTTCT-3′ and reverse 5′-TTGCATGTGAGGCTCTATCC-3′ (accession number NM_012238.4);* Homo sapiens* 18S ribosomal RNA (*18S*), forward 5′-GTAACCCGTTGAACCCCATT-3′ and reverse 5′-CCATCCAATCGGTAGTAGCG-3′ (accession number NR_003286);* Rattus norvegicus* tumor necrosis factor-alpha (*TNF-α*), forward 5′-TTCTCATTCCTGCTCGTGGC-3′ and reverse 5′-GCCCATTTGGGAACTTCTCCT-3′ (accession number NM_012675.3);* Rattus norvegicus* interleukin-1 beta (*IL-1β*), forward 5′-AGCTTTCGACAGTGAGGAGAA-3′ and reverse 5′-TCATCTGGACAGCCCAAGTC-3′ (accession number NM_031512.2);* Rattus norvegicus* inducible nitric oxide synthase (*iNOS*), forward 5′-AAAACCCCAGGTGCTATTCCC-3′ and reverse 5′-TCCAGGGATTCTGGAACATTCTGT-3′ (accession number NM_012611.3);* Rattus norvegicus* cyclooxygenase-2 (*COX-2*), forward 5′-TTCCAAACCAGCAGGCTCAT-3′ and reverse 5′-AAAAGCAGCTCTGGGTCGAA-3′ (accession number L20085.1);* Rattus norvegicus* sirtuin 1 (*SirT1*), forward 5′-AGGCCACGGATAGGTCCATA-3′ and reverse 5′-GAATTGTTCGAGGATCGGTGC-3′ (accession number XM_006223877.2);* Rattus norvegicus* 18S ribosomal RNA (*Rn18S*), forward 5′-CCTGGTTGATCCTGCCAGTAG-3′ and reverse 5′-GGCCGTGCGTACTTAGACAT-3′ (accession number NR_046237.1). Calculations of relative gene expression (normalized to 18S reference gene) were performed according to the ΔΔCT method. Fidelity of the PCR reaction was determined by melting temperature analysis.

### 2.7. Nuclear and Cytoplasmic Protein Extraction

To examine the effects of THSG in* P. gingivalis* LPS-induced NF-*κ*B activation, nuclear and cytoplasm proteins were extracted and isolated from HGFs by using of the Thermo Scientific NE-PER® Nuclear and Cytoplasmic Extraction Kit protocol (Thermo Scientific, Rockford, IL, USA). Briefly, HGFs were lysed in cytoplasm extraction reagent and were centrifuged at 16,000 ×g for extraction of the nuclear material. Proteins from the nuclear material were further extracted by the addition of nuclear extraction reagent to the nuclei and centrifuged at 16,000 ×g. Nuclear and cytoplasmic extracts were stored at −80°C until use. The protein concentration of the nuclear and cytoplasmic extracts was determined with the BCA*™* Protein Assay kit (Thermo Scientific).

### 2.8. Western Blot Analysis

To examine the effects of THSG in* P. gingivalis* LPS-induced NF-*κ*B activation in nucleus, we performed Western blot analysis to quantify the protein expression levels of NF-*κ*B in the nuclear extracts of HGFs which had been pretreated with 25 *μ*M THSG and followed by the treatment with 1 *μ*g/mL* P. gingivalis* LPS for 3 h. Nuclear protein samples were resolved on a 10% sodium dodecyl sulfate polyacrylamide gel (SDS-PAGE). A 20-*μ*g quantity of protein was loaded in each well with 5x sample buffer, and the protein samples were resolved by electrophoresis at 100 V for 2 hrs. The resolved proteins were transferred from the polyacrylamide gel to Millipore Immobilon-PSQ Transfer PVDF membranes (Millipore, Billerica, MA, USA) with the Mini Trans-Blot® Cell (Bio-Rad Laboratories, Inc., Hercules, CA, USA). The membranes were blocked with a solution of 2% fetal bovine serum in Tris-buffered saline. The membranes were incubated with primary antibodies to NF-*κ*B, phospho-p44/42 MAPK (pERK1/2), ERK2, glyceraldehyde 3-phosphate dehydrogenase (GAPDH) (GeneTex International Corporation, Hsinchu City, Taiwan), phospho-AMPK*α*, AMPK*α* (Cell Signaling Technology, Inc., Beverly, MA, USA), and Lamin B1 (Abcam, Cambridge, MA, USA), at 4°C overnight and washed, and the proteins were detected with HRP-conjugated secondary antibodies and Immobilon*™* Western HRP Substrate Luminol Reagent (Millipore). Images of the Western blots were visualized and recorded by BioSpectrum® Imaging System (UVP, LLC, Upland, CA, USA).

### 2.9. Ligature-Induced Experimental Periodontitis in Rats

All rats were housed in a reserved, pathogen-free facility and were handled in accordance with protocols approved by the Institutional Animal Care and Use Committee, National Defense Medical Center, Taipei, Taiwan (IACUC-15-361). A group of 8-week-old male Sprague-Dawley rats weighing between 200 and 250 g (*n* = 50) were randomly divided into eleven treatment groups defined as follows: nonligature (control, *n* = 10); ligature (*n* = 10); ligature + 0.1 mg/kg/d of THSG (*n* = 5); ligature + 10 mg/kg/d of THSG (*n* = 5); ligature + 25 mg/kg/d of resveratrol (*n* = 5); ligature + 12.5 mg/kg/d of ethanol extracts (*n* = 5); ligature + 25 mg/kg/d of ethanol extracts (*n* = 5); ligature + 50 mg/kg/d of ethanol extracts (*n* = 5). For the ligation, THSG, and ethanol extracts groups, experimental periodontitis was induced by placing 3-0 silk sutures around mandibular first molars at the gingival level (two molars in each rat), as described in our previous study [34]. The rats in the THSG and ethanol extracts groups also received these extracts by gavage feeding, starting 1 d before ligation. The rats in the control and ligature groups received sterile water daily. On day 8, all rats were sacrificed by CO_2_ inhalation, and the mandibles were excised and silk-surrounded gingiva was also collected for further mRNA examination, and then the mandibles were fixed in 4% paraformaldehyde. The mandibular specimens were prepared for dental radiography.

### 2.10. Radiographic Examination

To verify the level of bone loss for these treatment groups, the mandibular molars were examined by digital radiography using a computerized imaging system (Asahi Xspot; Asahi Roentgen Ind. Co., Ltd., Kyoto, Japan). The X-ray tube was operated at 70 kV, with a current of 6 mA, for 0.64 s, and the source-to-sensor distance was 50 cm. The images were processed using an image management system (INFINITT Dental PACS image system; INFINITT North America Inc., Phillipsburg, NJ, USA). We determined the dental alveolar bone level, defined as the distance from the cementoenamel junction (CEJ) to the most coronal level of the alveolar bone crest (CEJ-bone distance), using IMAGE J processing software (Image J software, National Institutes of Health, Bethesda, MD, USA). We also measured the radiographic periodontal bone-supporting ratio. The two parameters of the first mandibular molar were recorded along the mesial or distal root surfaces, respectively [34].

### 2.11. Statistical Analyses

In this study, all of the collected data of immunoblot and nucleotide densities were analyzed by IBM® SPSS® Statistics software version 19.0 (SPSS Inc., Chicago, IL, USA). Two-tail Student's *t-test* was conducted and considered significant at *p* values < 0.05 (*∗* or #), 0.005 (*∗∗* or ##), and 0.001 (*∗∗∗* or ###).

## 3. Results

### 3.1. Resveratrol and THSG Stimulate Cell Proliferation in HGFs

**Figure 1:**
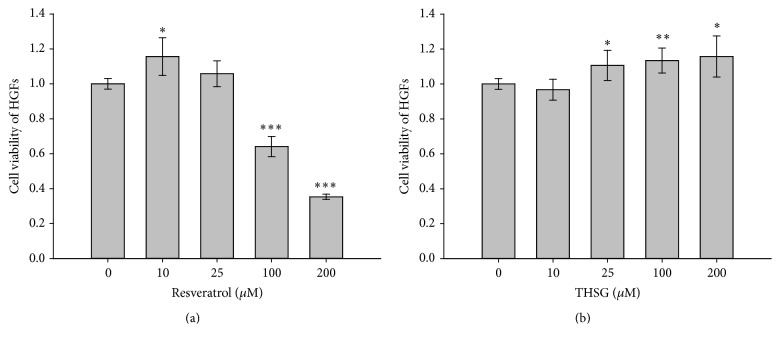
The cytotoxic effects of resveratrol and THSG in HGFs. HGFs (1 × 10^4^ cells/well) were cultured in 96-well plate and then were starved with serum-free DMEM/F12 medium for 24 h. After starvation, HGFs were treated with (a) resveratrol (0, 10, 25, 100, or 200 *μ*M) or (b) THSG (0, 10, 25, 100, or 200 *μ*M) in 0.25% stripped FBS medium for 72 h with reflashed medium with agents daily. (Numbers of experiments are 5, *N* = 5; ^*∗*^ *p* < 0.05; ^*∗∗*^ *p* < 0.01; ^*∗∗∗*^ *p* < 0.001 were compared with solvent/DMSO group.)

HGFs (1 × 10^4^ cells/well) were cultured in 96-well plate for 24 h and then starved with serum-free DMEM/F12 medium for 24 h. After starvation, HGFs were refed with medium containing 0.25% stripped FBS with different concentrations of resveratrol (0, 10, 25, 100, or 200 *μ*M) or THSG (0, 10, 25, 100, or 200 *μ*M) reflashed daily for 72 h. Resveratrol significantly enhanced cell proliferation of HGFs at low concentration (10 *μ*M) but inhibited cell proliferation at high concentrations (100 and 200 *μ*M) significantly (Figure 1(a)). On the other hand, THSG significantly enhanced HGFs cell proliferation when the concentration was over 25 *μ*M and did not show any cytotoxic effect in HGFs (Figure 1(b)).

### 3.2. Resveratrol and THSG Induce Anti-Inflammatory Effects in* P. gingivalis* LPS-Treated HGFs

**Figure 2:**
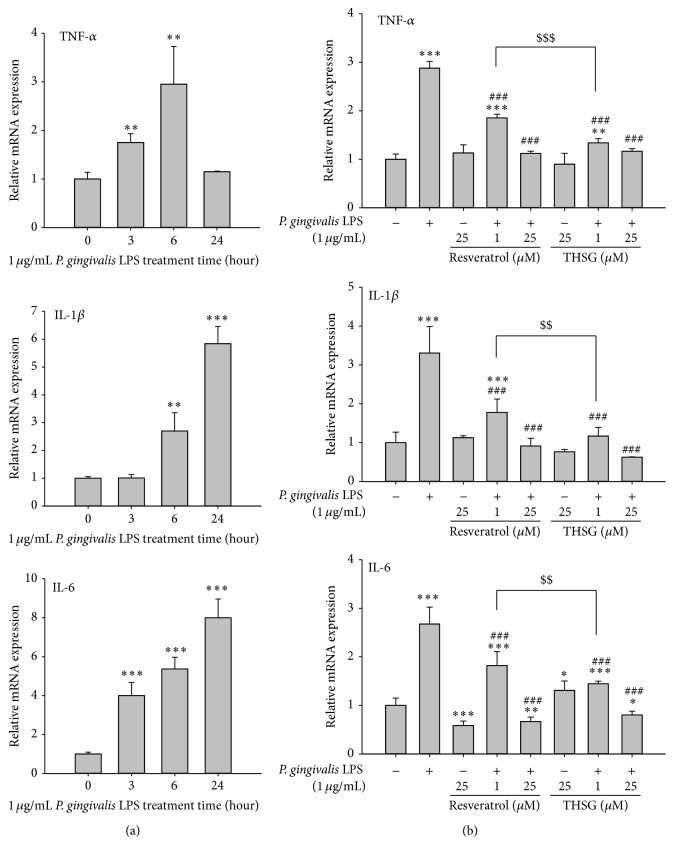
The anti-inflammatory effects of resveratrol and THSG in* P. gingivalis* LPS-treated HGFs. HGFs were cultured in 6-well plate and were starved with serum-free DMEM/F12 medium for 24 h when the cell confluence reached 80%. (a) HGFs were treated with 1 *μ*g/mL* P. gingivalis* LPS for 0, 3, 6, and 24 h. (b) HGFs were pretreated with resveratrol and THSG (1 and 25 *μ*M) for 30 min and then were treated with 1 *μ*g/mL* P. gingivalis* LPS for 6 h. Total RNA was extracted and reverse transcripted to cDNA, and then the quantitative PCR was performed. (*N* = 3; ^*∗*^ *p* < 0.05; ^*∗∗*^ *p* < 0.01; ^*∗∗∗*^ *p* < 0.001 were compared with control group. ^###^ *p* < 0.001 were compared with LPS group. ^$$^ *p* < 0.01; ^$$$^ *p* < 0.001 were compared with 1 *μ*M resveratrol group with LPS treatment.)

HGFs were cultured in 6-well plate. After starvation, cells were treated with 1 *μ*g/mL of LPS extracted from* P. gingivalis* for 0, 3, 6, and 24 h prior to harvest. Total RNA was extracted and quantitative PCR was performed. LPS induced the expression of TNF-*α* at 3 h and maximum expression at 6 h (Figure 2(a), upper panel). LPS stimulated the expression of IL-1*β* at 6 h and expressed more at 24 h. LPS stimulated the expression of IL-6 at 3 h, 6 h, and 24 h (Figure 2(a), middle and lower panel).

HGFs were cultured in 100 mm Petri dish and starved with serum-free DMEM/F12 medium for 24 h before studies. HGFs were pretreated with resveratrol or THSG (1 and 25 *μ*M) for 30 min and then treated with 1 *μ*g/mL* P. gingivalis* LPS for another 6 h. Resveratrol and THSG significantly attenuated LPS-induced expression of proinflammatory cytokines such as TNF-*α*, IL-1*β*, and IL-6 (Figure 2(b)). The inhibitory effect on the expression of proinflammatory cytokines by low concentration of THSG (1 *μ*M) was more than that of resveratrol.

### 3.3. THSG Attenuate* P. gingivalis *LPS-Induced Proinflammatory Cytokines Expression* via* Induced SirT1 Expression in HGFs

**Figure 3:**
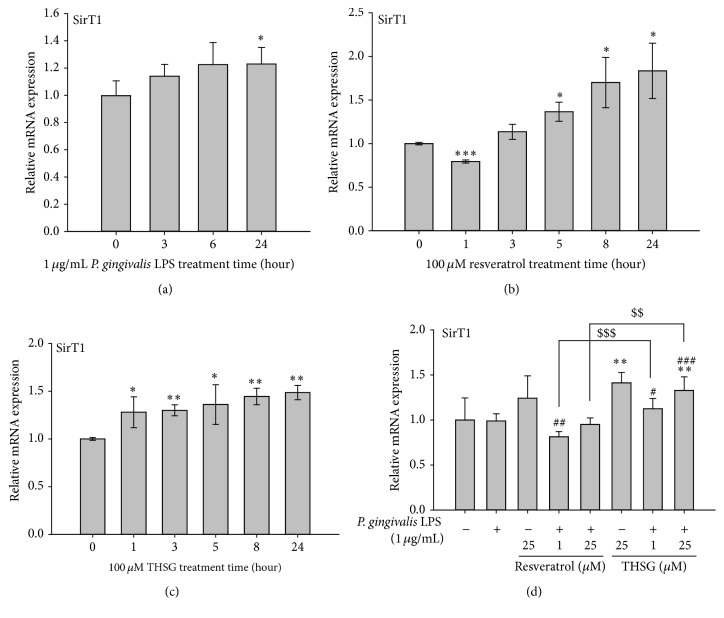
THSG attenuated* P. gingivalis* LPS-induced proinflammatory cytokines expression* via* inducing SirT1 expression in HGFs. HGFs were cultured in 6-well plate and were starved with serum-free DMEM/F12 medium for 24 h when the cell confluence reached 80%. (a) HGFs were treated with 1 *μ*g/mL* P. gingivalis* LPS, (b) 100 *μ*M resveratrol, and (c) 100 *μ*M THSG for 24 h. (d) HGFs were pretreated with resveratrol and THSG (1 and 25 *μ*M) for 30 min and then were treated with 1 *μ*g/mL* P. gingivalis* LPS for 6 h. The mRNA expression of SirT1 was examined by quantitative PCR. (*N* = 3;  ^*∗*^ *p* < 0.05; ^*∗∗*^ *p* < 0.01; ^*∗∗∗*^ *p* < 0.001 were compared with control group. ^#^ *p* < 0.05; ^##^ *p* < 0.01; ^###^ *p* < 0.001 were compared with LPS group. ^$$^ *p* < 0.01; ^$$$^ *p* < 0.001 were compared with 1 *μ*M or 25 *μ*M resveratrol group with LPS treatment.)

Activation of SirT1 is involved in anti-inflammatory process [16, 35]. It is of interest to investigate if activation of SirT1 also plays a role in THSG-inhibited proinflammatory cytokines expression. HGFs were cultured in 6-well plate and were starved with serum-free DMEM/F12 medium for 24 h when the cell confluence was reached to 80%. HGFs were treated with 1 *μ*g/mL of LPS extracted from* P. gingivalis*, 100 *μ*M resveratrol, or THSG, and then RNA was extracted. QPCR of SirT1 was conducted. Results shown in Figure 3(a) indicate that LPS significantly induced SirT1 expression at 24 h. However, resveratrol significantly reduced the mRNA expression of SirT1 at 1 h but enhanced it at 5 h to 24 h dramatically (Figure 3(b)). On the other hand, THSG constantly enhanced the mRNA expression of SirT1 significantly from 1 h to 24 h (Figure 3(c)). HGFs were pretreated with resveratrol or THSG (1 and 25 *μ*M) for 30 min and then treated with 1 *μ*g/mL* P. gingivalis* LPS for another 6 h. The mRNA expression of SirT1 was significantly enhanced by 25 *μ*M THSG but significantly reduced by 1 *μ*M resveratrol when treated with* P. gingivalis* LPS (Figure 3(d)). The enhanced effect on the expression of SirT1 by THSG was more than that of resveratrol when cells were treated with* P. gingivalis* LPS.

### 3.4. Signal Transduction Is Involved in THSG-Induced Anti-Inflammation

**Figure 4:**
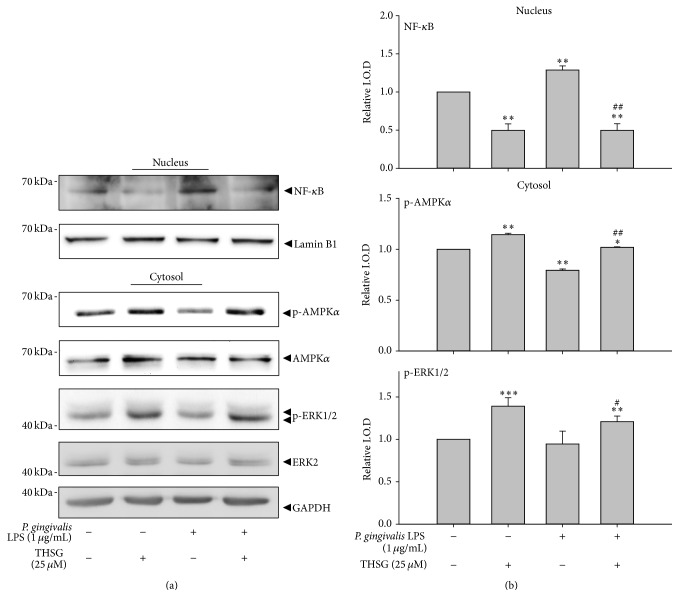
THSG attenuated* P. gingivalis* LPS-induced proinflammatory cytokines expression* via* reducing NF-*κ*B activation and activating AMPK and ERK1/2 in HGFs. HGFs were cultured in 10-cm dishes and starved for 24 h when the cell confluence reached 80%. HGFs were pretreated with 100 *μ*M resveratrol and THSG for 30 min and then treated with 1 *μ*g/mL* P. gingivalis* LPS for another 3 h. (a) The cytosolic and nuclear proteins of HGFs were extracted and Western blot was performed. (b) The relative intensity of nuclear translocation of NF-*κ*B and activation of AMPK and ERK1/2 were evaluated. (*N* = 3; ^*∗*^ *p* < 0.05; ^*∗∗*^ *p* < 0.01; ^*∗∗∗*^ *p* < 0.001 were compared with control group. ^#^ *p* < 0.05; ^##^ *p* < 0.01 were compared with LPS group.)

Signal transductions involved in the inhibitory effect of THSG on LPS-induced inflammation were also studied. HGFs were pretreated with resveratrol or THSG (25 *μ*M) for 30 min and then treated with 1 *μ*g/mL* P. gingivalis* LPS for another 3 h. The cytosolic and nuclear proteins of HGFs were extracted. In nuclear protein, the nuclear translocation of NF-*κ*B was significantly augmented by LPS in HGFs. However, THSG did not only significantly reduce LPS-augmented nuclear translocation of NF-*κ*B but also decrease that when THSG was presented alone in HGFs (Figures 4(a) and 4(b), upper panel). In cytosolic protein of HGFs, the activation of AMPK and ERK1/2 was significantly enhanced by THSG. On the other hand, treatment of LPS significantly reduced AMPK activation. Pretreatment of THSG significantly enhanced LPS-reduced AMPK activation and increased ERK1/2 activation even in the presence of LPS (Figures 4(a) and 4(b), middle and lower panel).

### 3.5. THSG Attenuates Ligature-Induced Experimental Periodontitis

**Figure 5:**
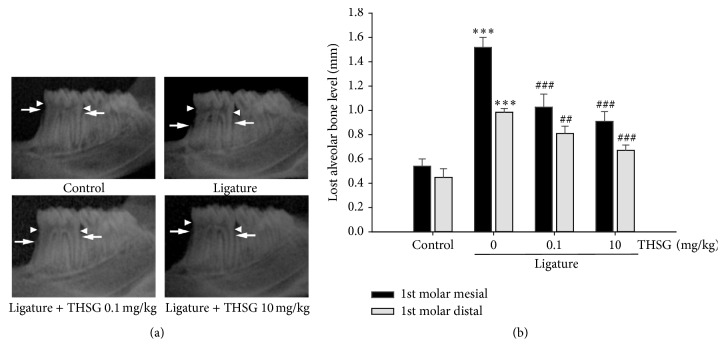
THSG attenuated ligature-induced experimental periodontitis. Rats were performed ligature-induced experimental periodontitis at first molar and fed with THSG (0.1 or 10 mg/kg/day dissolved in sterile water) or sterile water by gavage for 7 days. (a) The alveolar bone crest (white arrow) and the cementoenamel junction (CEJ, white arrowhead) were shown on radiographic images of the mandible from these treatments. (b) The radiographic lost alveolar bone level was measured along the mesial/distal root surface and was defined as the distance from the CEJ to the most coronal level of the alveolar bone crest. (^*∗∗∗*^ *p* < 0.001 was compared with control group. ^##^ *p* < 0.01; ^###^ *p* < 0.001 were compared with 0 mg/kg THSG group.)

Ligature-induced experimental periodontitis was performed in rats (8-week-old) at their first molar. Rats were fed with THSG (0.1 or 10 mg/kg/day dissolved in sterile water) or sterile water by gavage for 7 days. Radiographic films were taken and the lost alveolar bone level was evaluated (Figure 5(a)). The lost alveolar bone level was significantly increased in ligature group at first molar. However, administration of THSG significantly attenuated ligature-induced alveolar bone level lost (Figure 5(b)).

### 3.6. THSG Attenuates Proinflammatory Cytokines and Mediators mRNA Expression in the Gingival Tissues of Rats

**Figure 6:**
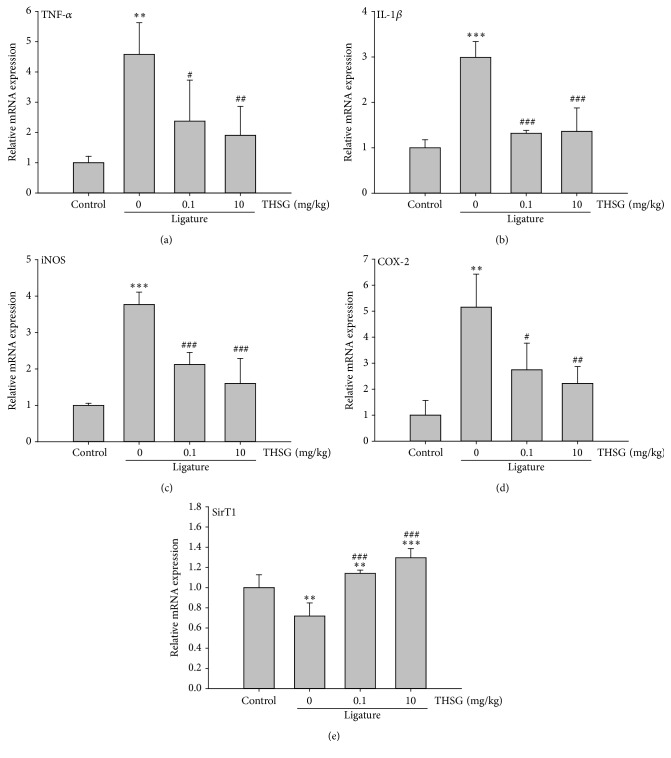
THSG attenuated the mRNA expression of proinflammatory cytokines and mediators in the gingival tissues of rats. Rat gingival tissues were collected from the first molars of mandible and homogenized. Their RNAs were extracted and reverse transcripted to cDNA. QPCR was performed to examine the mRNA expression of TNF-*α* (a), IL-1*β* (b), iNOS (c), COX-2 (d), and SirT1 (e). (^*∗∗*^ *p* < 0.01; ^*∗∗∗*^ *p* < 0.001 were compared with control group. ^#^ *p* < 0.05; ^##^ *p* < 0.01; ^###^ *p* < 0.001 were compared with 0 mg/kg THSG group.)

The mRNA expression of proinflammatory cytokines and mediators, including TNF-*α*, IL-1*β*, iNOS, COX-2, and SirT1, in rat gingival tissues was evaluated by QPCR. THSG significantly attenuated ligature-induced TNF-*α*, IL-1*β*, iNOS, and COX-2 mRNA expressions, even in low concentration (0.1 mg/kg) (Figures 6(a)–6(d)). In addition, administration of THSG significantly enhanced the mRNA expression of SirT1 in rat gingival tissues (Figure 6(e)).

### 3.7. P. Multiflori Ethanol Extract and THSG Have Similar Effect on the Amelioration of Ligature-Induced Experimental Periodontitis

**Figure 7:**
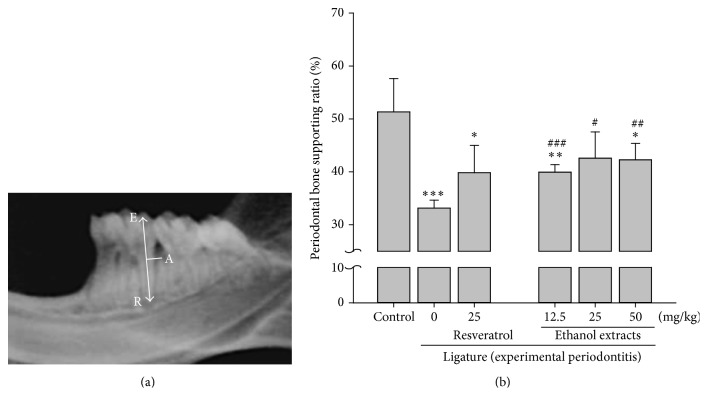
P. Multiflori ethanol extracts attenuated ligature-induced experimental periodontitis. Rats were performed ligature-induced experimental periodontitis at first molar and then fed with resveratrol (25 mg/kg/day dissolved in 50% ethanol), ethanol extracts (12.5, 25, or 50 mg/kg/day dissolved in 50% ethanol), or 50% ethanol by gavage for 7 days. (a) The periodontal bone-supporting ratio was measured along the distal root surface and was labeled (based on measurements spanning the cusp tip, E, the deepest bony defect, A, and the root apex, R) as the A-R distance divided by the E-R distance × 100%. (b) The periodontal bone-supporting ratio was measured and shown. (^*∗*^ *p* < 0.05; ^*∗∗*^ *p* < 0.01; ^*∗∗∗*^ *p* < 0.001 were compared with control group. ^#^ *p* < 0.05; ^##^ *p* < 0.01; ^###^ *p* < 0.001 were compared with ligature group.)

To evaluate whether the crude extracts of P. Multiflori would also have ameliorated effect on periodontitis, ligature-induced experimental periodontitis was performed in rats at their first molar and rats were fed with resveratrol (25 mg/kg/day), ethanol extracts (12.5, 25, or 50 mg/kg/day), or 50% ethanol by gavage for 7 days. According to our previous reports [29, 34], the periodontal bone-supporting ratio was measured. The radiographic image demonstrated the mandible from a ligatured rat and the measurements used to measure the periodontal bone-supporting ratio (Figure 7(a)). The periodontal bone-supporting ratio was significantly reduced in ligature group at first molar. Ethanol extracts of P. Multiflori significantly ameliorated ligature-induced periodontal bone-supporting ratio in all concentrations (Figure 7(b)). Damage of periodontal bone in rats receiving 25 mg/kg resveratrol was also reduced but there was no significant difference between resveratrol-treated group and control.

## 4. Discussion

Among all herb medicines, resveratrol contributes the most in terms of health benefits due to its antioxidant properties. It has been described as a scavenger of superoxides, hydroxyl radicals, and peroxynitrites [12]. Furthermore, resveratrol has been shown to induce activation of antioxidant enzymes [36, 37] and activates the nuclear factor E2-related factor (Nrf2) antioxidant defense pathway [38]. Activated Nrf2 translocates to the nucleus and mediates the transcription of target genes such as heme oxygenase 1 (HO-1) and NAD(P)H:quinine oxidoreductase 1 (NQO-1) which are involved in cellular resistance to oxidative stress which confers protection against inflammation [39].

When human gingival fibroblasts were treated with different concentrations of resveratrol or THSG, resveratrol only stimulated cell proliferation at low concentration (Figure 1) but THSG stimulated cell proliferation at all concentrations (1 to 250 *μ*M) examined (Figure 1). These results suggest that there is less cytotoxic of THSG than of resveratrol. Resveratrol suppressed* P. gingivalis* LPS-stimulated I*κ*B*α* phosphorylation and nuclear translocation of the p65 subunit of NF*κ*B in HMECs [21]. The concentration of IL-17 in the resveratrol group is lower than that in control group gingival tissue (*p* < 0.05); however, there is no difference in the IL-1*β* and IL-4 levels of the groups (*p* > 0.05) [40]. THSG activated AMPK and ERK1/2 as resveratrol (Figure 2). In addition, both resveratrol and THSG inhibited NF*κ*B activation which is essential for the expression of inflammatory cytokines.

Resveratrol is also able to downregulate the cell adhesion molecules, ICAM-1 and VCAM-1, and significantly inhibits the* P. gingivalis* LPS-induced adhesion of leukocytes to endothelial cells and to the aortic endothelium [21]. In addition, resveratrol is able to reduce the inducible NO synthase (iNOS) expression [20] and further prevents NO production. This characteristic contributes to oxidative stress reduction to decrease the systemic levels of some proinflammatory cytokines and result in resveratrol's immunomodulatory effect [40, 41].

In addition, resveratrol can stimulate mitochondrial biogenesis by increasing the expression of NAD-dependent nuclear class III histone deacetylase, sirtuin 1 (SirT1)* via* the activated AMP-activated protein kinase (AMPK) [42, 43]. Our studies also indicate that THSG and resveratrol were able to activate SirT1 and showed a role in the inhibitory effect on LPS-induced expression of inflammatory cytokines (Figure 3(b)).

Both ethanol extract THSG and pure compound did inhibit ligature-induced periodontitis (Figures 5 and 7). On the other hand, the treatment of resveratrol did not attenuate ligature-induced periodontitis of rats (*p* = 0.054) in our studies (Figure 7). Studies conducted by Casati et al. using 10 mg/kg resveratrol per day for 19 days pretreatment indicated that there were higher bone loss values in ligated molars and unligated teeth in the control group than in the resveratrol-fed group (*p* < 0.05) [40]. Resveratrol added in drinking water has been shown to lead to relieve ligature placement associated alveolar bone resorption [41]. Further, systemic administration of resveratrol continuously also decreased ligature-induced periodontal breakdown [40]. Those studies and our results confirm the positive effects of resveratrol on ligature-induced periodontitis animal model. Studies have also found a powerful effect of resveratrol in preventing lipid peroxidation, specifically low density lipoprotein (LDL) [44]. Improved endothelial function and enhanced cardioprotective effects have been observed with resveratrol through its anti-inflammatory and antioxidant properties in various animal models of myocardial injury, hypertension, and type 2 diabetes [39, 45, 46].

In summary, THSG and resveratrol prevented the development of ligature-induced rat modeling periodontitis. Similar to resveratrol, THSG activated the expression of SirT1 during the process. Although both resveratrol and THSG activated ERK1/2 and AMPK and inhibited NF*κ*B activation in human gingival fibroblasts, regenerating capacity of THSG is better than that of resveratrol. THSG and resveratrol both inhibited the expression of proinflammatory cytokines such as IL-1*β* and TNF-*α*, which are involved in pathogenesis of periodontitis. Yet again, the efficiency of THSG on the prevention of periodontitis is better than that of resveratrol. From all those evidences, it suggests that THSG either in crude extract or as pure compound may be used in the prevention and treatment of periodontitis in the future.
